# A pilot bedtime routine intervention for toddlers in primary care: variation by caregiver educational attainment

**DOI:** 10.3389/frsle.2025.1722530

**Published:** 2026-01-06

**Authors:** Jodi A. Mindell, Joey Tsz Ying Lam, Zainab Salih, Megan Heere, Ariel A. Williamson

**Affiliations:** 1 Department of Psychology, Saint Joseph's University, Philadelphia, PA, United States; 2 Division of Pulmonary and Sleep Medicine, Children's Hospital of Philadelphia, Philadelphia, PA, United States; 3 College of Education, Lehigh University, Bethlehem, PA, United States; 4 Department of Pediatrics, Lewis Katz School of Medicine, Temple University, Philadelphia, PA, United States; 5 The Ballmer Institute for Children's Behavioral Health, The University of Oregon, Portland, OR, United States

**Keywords:** bedtime routines, social-emotional development, behavioral intervention, education, toddlers

## Abstract

**Background:**

A consistent bedtime routine (≥5 nights per week) is an empirically supported intervention associated with better sleep outcomes. However, few studies have examined the impacts of a bedtime routine on outcomes beyond sleep, and among families of lower educational attainment.

**Objective:**

This pilot randomized controlled trial (RCT) examined initial outcomes (sleep, development, caregiver stress), feasibility, and acceptability of a primary care-based bedtime routine intervention for toddlers, and explored variation in outcomes by caregiver educational attainment.

**Method:**

Caregivers of 86 toddlers (*M*_age_ = 12.89 months, 67.4% Black/African American, 23.3% Hispanic/Latine; United States) were randomly assigned to a bedtime routine intervention or usual care at their 12-month well-child visit (age-based preventative care). At their 15- and 24-month well visits, child sleep (Brief Infant Sleep Questionnaire–R SF), social-emotional development (Brief Infant-Toddler Social and Emotional Assessment), caregiver stress (Parenting Stress Inventory-SF), and intervention acceptability were assessed.

**Results:**

There were no differences in outcomes between the groups, however, the intervention positively impacted sleep consolidation, social-emotional outcomes, and caregiver stress, primarily at 24 months of age, for toddlers of caregivers with lower educational attainment. Additionally, families in the intervention were more likely to include reading in their bedtime routine at 15 months. Caregivers assigned to the intervention also reported strong acceptability and 85% completed both sessions.

**Conclusions:**

This pilot study suggests that bedtime routine intervention for toddlers is acceptable, feasible, and results in increased integration of reading at 15 months of age. Caregivers of lower educational attainment in the intervention condition reported improvements in aspects of child sleep health, social-emotional concerns, and caregiver stress, highlighting the potential for this intervention to reduce sleep health disparities. Future research should continue to examine potential bedtime routine benefits beyond sleep in larger-scale RCTs.

## Introduction

Healthy sleep in young children is associated with positive cognitive, language, and motor development, as well as emotional regulation (Maski and Kothare, 2013; Morales-Munoz et al., 2020). A consistent bedtime routine (five or more nights per week) is associated with better sleep outcomes and is an empirically supported intervention (Meltzer et al., 2021a; Mindell et al., 2009; Mindell and Williamson, 2018). In addition to sleep benefits, bedtime routines are hypothesized to foster caregiver-child attachment, health behaviors, prosocial development, and social-emotional development (Mindell and Williamson, 2018). However, few studies have investigated the additional potential benefits of bedtime routines (Kelly et al., 2013; Ren and Hu, 2019; Zajicek-Farber et al., 2014) or the benefits of bedtime routines in the context of socioeconomic factors that are linked to bedtime routine consistency as well as broader pediatric sleep health disparities (Jackson et al., 2020; Schwichtenberg et al., 2019).

Lower caregiver educational attainment is one socioeconomic factor linked to less consistent bedtime routine implementation in prior research (Hale et al., 2009; Williamson and Mindell, 2020). Recent work also indicates that caregiver educational attainment may moderate the link between bedtime routine consistency and socio-emotional outcomes (Lam et al., 2023). Bedtime routine implementation and other pediatric sleep interventions are rarely tested among families of lower socioeconomic status (SES) and/or lower educational attainment (Schwichtenberg et al., 2019), or in real-world service delivery contexts, such as primary care. While it is important to assess the outcomes of a bedtime routine intervention on sleep, socioemotional development, and parental functioning, especially during the key period of early childhood development (Black et al., 2017), it is also crucial to identify moderating socioeconomic factors and test interventions in the settings where they can ultimately be scaled. These approaches can help address sleep health disparities and identify for whom interventions are most efficacious (Baumann and Cabassa, 2020; Jackson et al., 2020; Weisz, 2014).

### Bedtime routines and outcomes

Bedtime routines include a consistent set of activities, such as reading books, brushing teeth, and bathing, that occur every night in the hour before lights out (Mindell and Williamson, 2018). Furthermore, bedtime routine activities can be categorized into a number of components, including nutrition, hygiene, communication, and physical contact, which can promote positive outcomes across developmental domains, including sleep, health, literacy, and attachment, among others. The associated sleep benefits of implementing a consistent bedtime routine (i.e., five or more nights per week) with sleep outcomes are well-documented, including an earlier bedtime, decreased sleep onset latency, decreased number and duration of night wakings, increased nighttime sleep duration, and fewer caregiver-reported sleep problems (Fiese et al., 2021; Mindell et al., 2015; Mindell and Williamson, 2018; Shetty et al., 2022). Implementation of a bedtime routine is also effective in improving sleep outcomes, although it has only been investigated in two studies to our knowledge. One study of 405 infants and toddlers experiencing sleep problems who were randomized to either a nightly bedtime routine or control group, found improvements in multiple aspects of sleep, especially wakefulness after sleep onset and sleep continuity following institution of a consistent bedtime routine (Mindell et al., 2009). Another proof of concept study of 1- to 3-year-olds found a bedtime routine intervention resulted in increased sleep duration and less disrupted sleep compared to baseline, although there was no control group comparison (Kitsaras et al., 2022). Both of these studies included community-recruited samples of primarily White and/or higher-SES backgrounds (Schwichtenberg et al., 2019), with no studies conducted in primary care settings (Honaker and Meltzer, 2016).

A consistent bedtime routine is also expected to be associated with better overall development. For example, institution of a language-based bedtime routine is associated with better language ability (Hale et al., 2011), cognitive-academic skills, and subsequent academic attainment (Câmara-Costa et al., 2021). Having a consistent bedtime routine is additionally associated with better executive functioning and school readiness (Kitsaras et al., 2018), as well as social-emotional development (Lam et al., 2023; Ren and Hu, 2019; Zajicek-Farber et al., 2014). Furthermore, a consistent bedtime routine is postulated to improve family factors, such as caregiver stress (Mindell and Williamson, 2018), but few studies have examined these outcomes. None of these studies evaluated the connection between bedtime routine institution and development as part of a clinical trial.

### Bedtime routines and sleep health disparities

Several studies have found that children living in lower SES contexts are less likely to have a consistent bedtime routine (Covington et al., 2021). For example, in one study of 118 young children (12–47 mos), housing and food insecurity were associated with decreased likelihood of implementing a nightly bedtime routine (Covington et al., 2019). In another study of 3,217 3-year-olds, low maternal education, increased household size, and living in poverty were associated with decreased use of parent-child interactive and hygiene-related bedtime routines (Hale et al., 2009). These socioeconomic factors were linked to bedtime routine implementation and characteristics beyond differences across racial and ethnic groups. Variation in bedtime routine implementation by family socioeconomic characteristics may have implications for sleep and other bedtime-routine related child outcomes. For example, we previously found a significant interaction between bedtime routine consistency and caregiver's education attainment for future internalizing problems in young children (Lam et al., 2023). Specifically, toddlers whose caregivers had a high school education or less and lacked a consistent bedtime routine at 15 months displayed more internalizing problems at 24 months.

### Current study

Overall, there has been minimal research conducted on the efficacy of a bedtime routine intervention on sleep outcomes with little focus on other aspects of development, especially during toddlerhood, a critical period of development. Furthermore, it is important to consider multilevel social and environmental contributors that may help focus for whom a particular intervention is most efficacious (Covington, 2023; Jackson et al., 2020). Family educational attainment, a socioeconomic factor, has been shown to moderate the relationship between sleep patterns and behaviors (i.e., bedtime routines) and socio-emotional outcomes (El-Sheikh et al., 2022; Lam et al., 2023; Williamson et al., 2021; Williamson and Mindell, 2020). Understanding moderating factors in the context of intervention effects is also necessary for future research tailoring interventions to better address socioeconomic sleep health disparities (Baumann and Cabassa, 2020; Jackson et al., 2020; Weisz, 2014).

Pilot testing interventions with populations experiencing health disparities (e.g., caregivers with lower educational attainment) and in the settings for which they are ultimately designed (i.e., primary care, a context that is accessible for most families with young children) is crucial for ensuring feasibility and preventing additional disparities when interventions are tested on a larger scale. Thus, the current randomized controlled trial (RCT) was designed as a pilot project to assess primary child sleep and secondary developmental outcomes, including caregiver stress and intervention feasibility and acceptability, of a bedtime routine intervention for toddlers implemented in an outpatient primary care site serving a population with varying levels of caregiver educational attainment. This pilot RCT compared a bedtime routine intervention, which was provided at 12- and 15-month well visits (scheduled age-based preventative care), to usual care in the primary care site, with outcomes assessed at 15- and 24-month well visits. We hypothesized that, compared to usual pediatric care, a bedtime routine intervention would be associated with better sleep outcomes (primary aim) and social-emotional development in toddlers, as well as reduced caregiver stress, at 15- and 24-month well visits (secondary aim). Consistent with the aims and scope of a pilot trial (Leon et al., 2011; Teresi et al., 2022), we also examined intervention feasibility and acceptability to inform future research (secondary aim). In addition, we explored whether bedtime routine intervention effects varied according to caregiver educational attainment, to better understand bedtime routine interventions in those with lower educational attainment (exploratory aim).

## Method

### Overview of design and setting

This pilot study was conducted in one urban (i.e., large, metropolitan) pediatric primary care office in Philadelphia, PA. Data were collected during the coronavirus 2019 (COVID-19) pandemic, from October 2020 to November 2022. Due to the impact of COVID-19 on family and clinician wellbeing and well visit scheduling, the study design and procedures, including the sample size, randomization procedure, inclusion criteria, and assessment schedule, were developed in partnership with the primary care site to maximize feasibility and minimize family and clinician burden.

Families were recruited at their child's 12-month well visit and assigned 1:1 to the intervention or usual care group based on the day of the week for their scheduled visit, such that intervention and usual care recruitment days occurred on two selected days of the week. Days were selected based on when the majority of well-child visits were typically scheduled and research assistant availability. The days selected were randomly assigned to either intervention or control, and then switched partway through the study to reduce day of the week recruitment bias. Primary care staff were kept blind to which days were treatment vs. control. The primary care site preferred this randomization option (i.e., 2 days per week) over family-level randomization, to reduce the number of staff present within their office during the COVID-19 pandemic. All families provided informed consent (separate for control and intervention groups) prior to completion of surveys.

At the 12-month visit, families completed a very brief baseline survey that only consisted of sociodemographic questions and questions about their child's bedtime routine. This approach was developed in partnership with the primary care site leadership to minimize family burden. When families returned for their child's 15- and 24-month well visit, caregivers completed questions about their child's bedtime routine as well as a more comprehensive set of measures, including questionnaires about child sleep, measures of social-emotional development and parenting stress, and intervention acceptability (intervention families only). Caregivers were not compensated for their participation in this study; this decision was made in collaboration with the primary care site leadership. This study was approved by a university Institutional Review Board and registered before the enrollment of the first participant at ClinicalTrials.gov (NCT04592172).

### Participants

**Figure 1:**
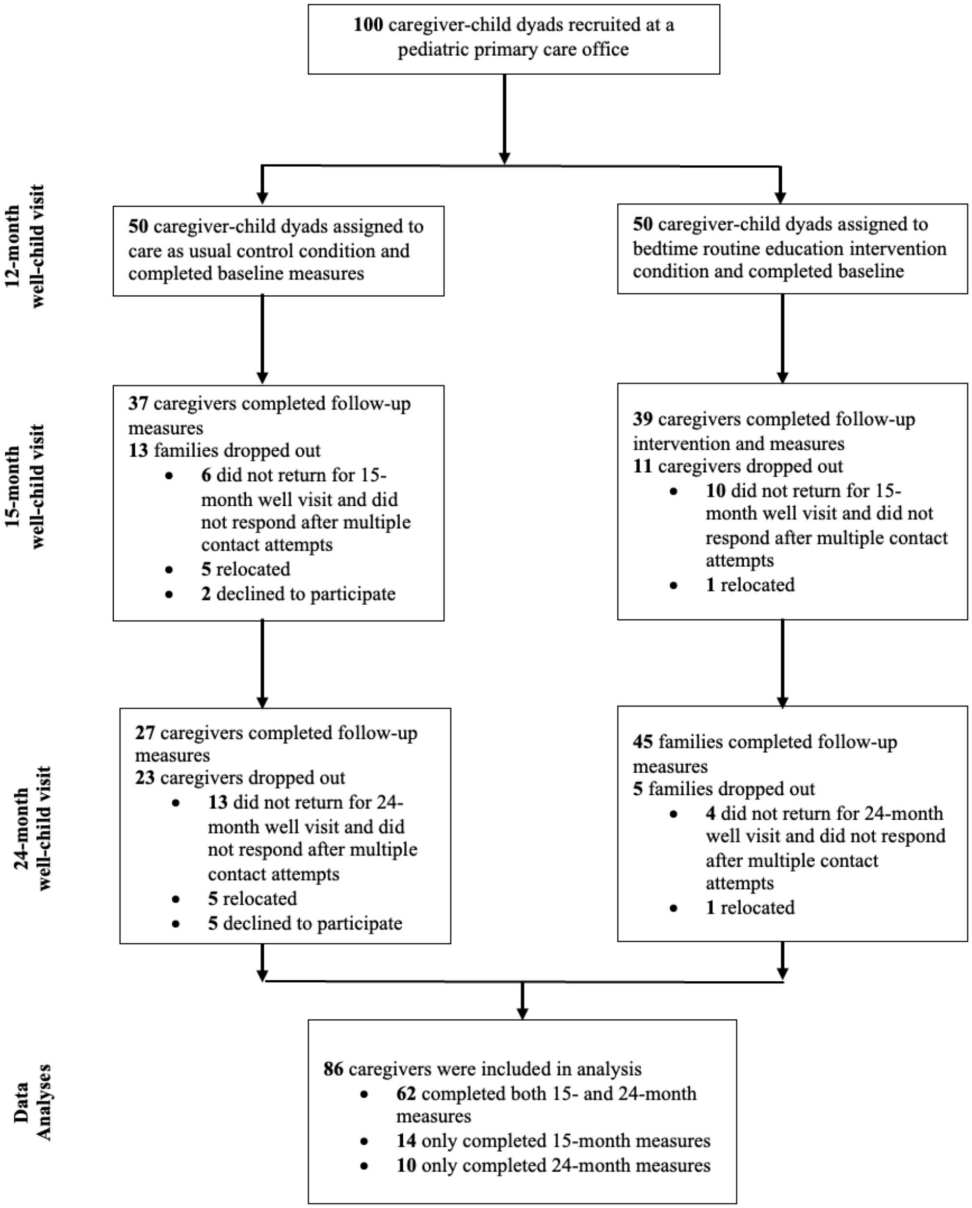
CONSORT flowchart of participants. Only bedtime routine frequency was assessed at 12 months.

One hundred families were recruited at their toddler's 12-month well visit. Inclusion criteria were (1) child age 12.0–14.9 months (2) primary caregiver or legal-guardian present, and (3) English-speaking. As shown in Figure 1, 100 families were recruited and randomly assigned to intervention (*n* = 50, 50.0%) and usual care (*n* = 50, 50.0%). Of these, 76 (76.0%) completed the 15-month questionnaires and 72 (72.0%) completed follow-up questionnaires. A total of 86 families, 46 of whom (53.3%) were assigned to intervention, completed baseline and the 15- and/or 24-month surveys, and were included in study analyses. Of these, 62 (72%) completed follow-up questionnaires at both their 15- and 24-month visits, 14 (16%) at their 15-month visit only, and 10 (12 %) at their 24-month visit only.

Sociodemographic information for the final sample of 86 caregivers (95.3% parents; 81.4% female) and toddlers (M_age_ = 12.89 months; 57.0% girls) are summarized in Table 1. Caregivers reported that 65.1% of toddlers were Black/African American, 24.4% Hispanic/Latine, and 9.3% White. Caregivers identified their race and ethnicity as 67.4% Black/African American, 23.3% Hispanic/Latine, and 9.3% White. Regarding educational attainment, 50.0% had a high school education or less. Sociodemographic information for the sample aligns with US Census estimates (https://data.census.gov/) for the zip code where the primary care site was located, with most residents identifying as Black/African American or Hispanic/Latine and 40.6% having a high school education or less. There were no differences between the intervention and usual care groups across any of these sociodemographic factors, *p* > 0.05.

**Table 1:** Sociodemographics.

**Sociodemographic information**	**Mean (SD); % (** ***n*** **)**		
	**Full sample**	**Intervention (*****n*** = **46)**	**Usual care (*****n*** = **40)**
**Child age (months)**			
12-month visit (range 12.03–14.87)	12.89 (0.83)	12.92 (0.85)	12.87 (0.82)
15-month visit (range 13.42–19.84)	16.52 (1.32)	16.92 (2.43)	16.59 (1.31)
24-month visit (range 23.23–30.06)	25.91 (1.55)	25.82 (1.47)	26.06 (1.69)
**Child sex**			
Boy	43.0 (37)	43.5 (20)	42.5 (17)
Girl	57.0 (49)	56.5 (26)	57.5 (23)
**Child race and ethnicity** ^*^			
Black/African American	65.1 (56)	67.4 (31)	60.0 (24)
Hispanic or Latine	24.4 (21)	21.7 (10)	27.5 (11)
White	9.3 (8)	6.5 (3)	12.5 (5)
WIC recipient	72.1 (62)	76.1 (35)	67.5 (27)
**Caregiver age**			
18–24	29.1 (25)	32.6 (15)	25.0 (10)
25–29	27.9 (34)	21.7 (10)	35.0 (14)
30–39	34.9 (30)	34.8 (16)	35.0 (14)
40–49	5.8 (5)	8.7 (4)	2.5 (1)
50+	2.3 (2)	2.2 (1)	2.5 (1)
**Caregiver sex**			
Male	18.6 (16)	17.4 (8)	20.0 (8)
Female	81.4 (70)	82.6 (38)	80.0 (32)
**Caregiver race and ethnicity** ^*^			
Black/African American	67.4 (58)	69.6 (32)	65.0 (26)
Hispanic or Latine	23.3 (20)	19.6 (9)	27.5 (11)
White	9.3 (8)	8.7 (4)	10.0 (4)
**Caregiver relation**			
Parent	95.3 (82)	95.7 (44)	95.0 (38)
Grandparent	2.3 (2)	2.2 (1)	2.5 (1)
Older sister	2.3 (2)	2.2 (1)	2.5 (1)
**Income**			
No income	16.3 (14)	10.9 (5)	22.5 (9)
$1–$20,000	12.8 (11)	15.2 (7)	10.0 (4)
$20,001–$35,000	10.5 (9)	10.9 (5)	10.0 (4)
$35,001–$50,000	7.0 (6)	10.9 (5)	2.5 (1)
$50,001–$75,000	4.7 (4)	4.3 (2)	5.0 (2)
$75,001–$100,000	2.3 (2)	0.0 (0)	5.0 (2)
$100,001 or more	2.3 (2)	2.2 (1)	2.5 (1)
Prefer not to answer	44.2 (38)	45.7 (21)	42.5 (17)
**Caregiver education**			
High school/secondary school	51.2 (44)	60.9 (28)	40.0 (16)
Diploma/pre-university/junior college	3.5 (3)	0.0 (0)	7.5 (3)
Some college/university	22.1 (19)	19.6 (9)	25.0 (10)
College/university	20.9 (18)	17.4 (8)	25.0 (10)
Postgraduate	2.3 (2)	2.2 (1)	2.5 (1)
**Marital status**			
Single, never married	50.0 (43)	54.3 (25)	45.0 (18)
Single, previously married	1.2 (1)	0.0 (0)	2.5 (1)
Married	18.6 (16)	17.4 (8)	20.0 (8)
Unmarried, living with partner	30.2 (26)	28.3 (13)	32.5 (13)
**Occupation status**			
Employed full-time	39.5 (34)	39.1 (18)	40.0 (16)
Employed part-time	15.1 (13)	13.0 (6)	17.5 (7)
Homemaker/at-home parent/on maternity leave	29.1 (25)	34.8 (16)	22.5 (9)
Student	2.3 (2)	0.0 (0)	5.0 (2)
Unemployed/between jobs	14.0 (12)	13.0 (6)	15.0 (6)

^*^Race and ethnicity are included as socio-political constructs, not biological indicators; WIC, Special Supplemental Nutrition Program for Women, Infants, and Children.

### Intervention procedures

#### Intervention condition

**Figure 2:**
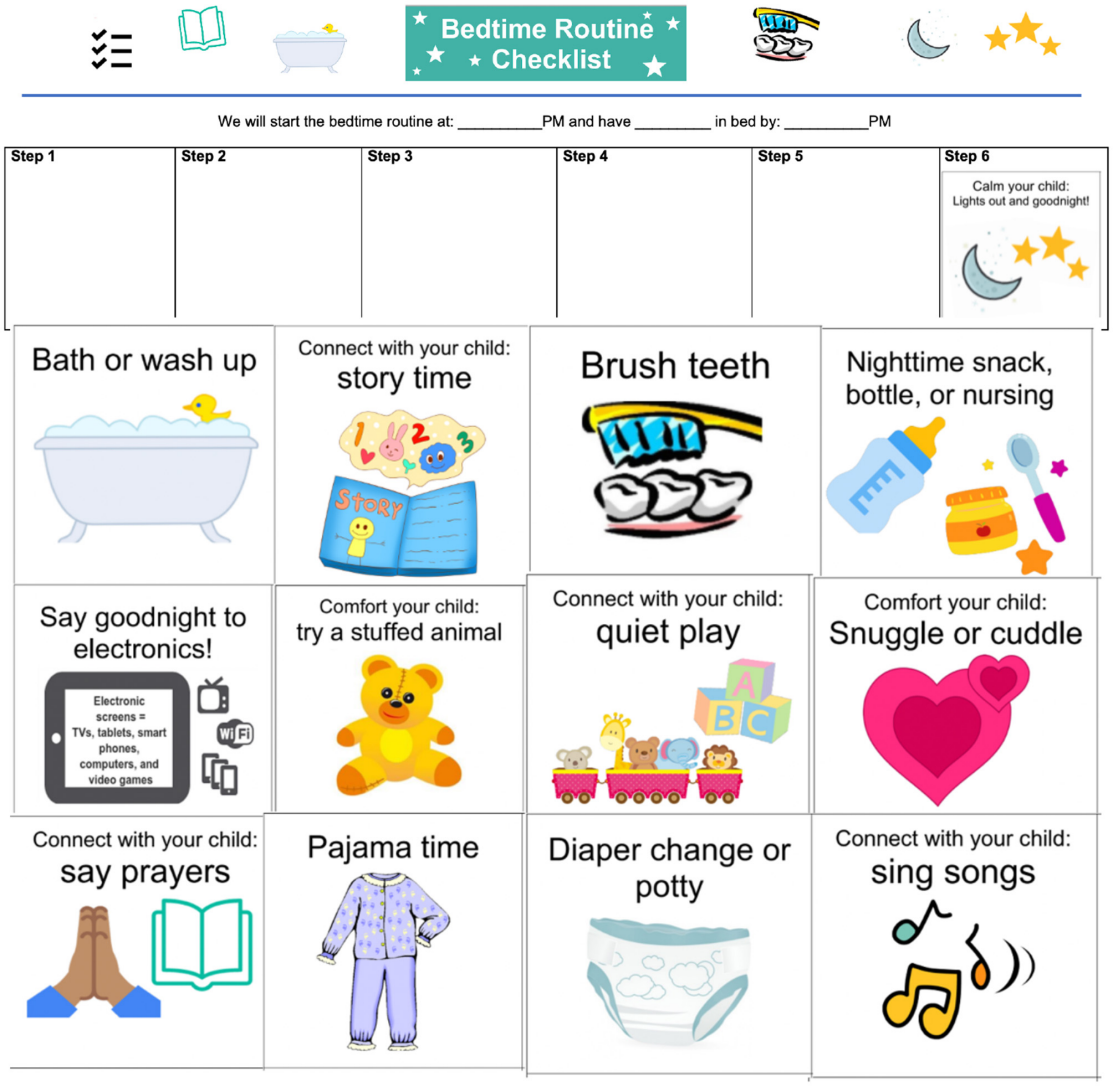
Bedtime routine intervention graphics and individualized chart.

The bedtime routine intervention (“3 Cs for Bedtime ZZZs” program) was named and organized around evidence-based bedtime routine components: Connect, to reflect caregiver-child interaction during routine activities; Comfort, to reflect cuddling and soothing before bedtime; and Calm, to reflect the transition to lights out and sleep. Bedtime routine materials, including an infographic (Figure 2), were drawn from a prior primary care-based sleep intervention (Williamson et al., 2022). At their child's 12-month visit, caregivers in the intervention group met with a trained research assistant, who provided the family with evidence-based education about the importance of bedtime routines in early childhood (Meltzer et al., 2021b; Mindell and Williamson, 2018) and then collaboratively worked with the family to develop an individualized toddler bedtime routine. The individualized routine included a bath, teeth brushing, and reading for all toddlers, as well as up to two additional activities of the family's choice (e.g., lullabies, prayers). A bedtime routine chart was created based on the chosen activities, which included pictograms of each activity (Figure 2). Families were provided with bath and lotion products, a toothbrush and toothpaste, and two age-appropriate books (in addition to a book provided at all well-child visits through *Reach Out and Read*, a national non-profit that supports pediatric literacy by encouraging shared reading and providing books to families of young children at well-child visits). They were also given a parenting toolkit, including educational information from the Simms/Mann Family Foundation about positive parenting interactions, a board book, and a stuffed animal. The family then met with trained research assistant again at the 15-month well visit to review their individualized routine, and were provided with additional products and books. Thus, the intervention occurred at both 12- and 15-months well visits.

All research assistants implementing the intervention were trained by the primary investigators (JAM, AAW), who are licensed psychologists specializing in sleep disorders. One of the investigators conducted fidelity checks for each research assistant by joining intervention sessions.

#### Usual care condition

The usual care condition reflected typical early childhood pediatrician practice, with families instructed to meet with their child's pediatrician for 15- and 24-month well visits, consistent with routine care.

### Baseline assessment

#### Sociodemographic information

Sociodemographic information was collected at the 12-month well visit, including caregiver's age, relationship to child, marital and employment status, education level, household income, and Pennsylvania Special Supplemental Nutrition Program for Women, Infants, and Children (WIC) enrollment status. In addition, racial and ethnic backgrounds for the caregiver and child were obtained. As in previous research (Hale et al., 2009; Lam et al., 2023), lower caregiver educational attainment was defined as having a high school education or less.

#### Bedtime routines

Caregivers completed one item from the Brief Infant Sleep Questionnaire–Revised Short Form (BISQ-R SF; Mindell et al., 2019) to indicate the number of nights per week their child followed the same bedtime routine (i.e., bedtime routine frequency). In line with prior research, a consistent bedtime routine was defined as having a bedtime routine for five or more nights per week (Mindell and Williamson, 2018; Williamson et al., 2020).

### Assessments at 15- and 24-month visits

#### Primary sleep outcomes

At 15- and 24-month visits, caregivers reported on their child's sleep for the past two weeks using the BISQ-R-SF. The BISQ-R SF is adapted from the well-validated Brief Infant Sleep Questionnaire (Sadeh, 2004), which has strong test-retest reliability and good correspondence with actigraphy-measured sleep and daily sleep logs. Sleep outcomes included sleep patterns (e.g., sleep onset latency, number and duration of night wakings, nighttime and daytime sleep that was summed to report total 24-h sleep duration) and caregiver perceptions (e.g., perception of sleep problems). Caregivers also responded to the bedtime routine frequency item, as noted above, and indicated the activities included in their child's bedtime routine.

#### Secondary social-emotional outcomes

Caregivers completed the 42-item Brief Infant-Toddler Social and Emotional Assessment (BITSEA; Briggs-Gowan et al., 2004) at their child's 15- and 24-month well visit to assess toddlers' social-emotional problems Child behaviors were rated based on the past month on a three-point Likert scale (0 = not true/rarely, 1 = somewhat true/sometimes, 2 = very true/often). This study examined the externalizing problems, internalizing problems, dysregulation, and maladaptive behaviors subscales, which are part of the broader 31-item BITSEA problem scale. These subscales were selected to reflect clinically meaningful indices of social and emotional functioning previously associated with early childhood sleep (Deng et al., 2023; Jung and Jin, 2019; Sivertsen et al., 2015). The BITSEA demonstrates excellent test-retest reliability (*r* = 0.82–0.92) and internal consistency (α = 0.65–0.79; Briggs-Gowan et al., 2004).

#### Secondary caregiver stress outcome

The Parenting Stress Index–Short Form (Abidin, 2012) was used to measure total caregiver stress. The 36-item measure includes assessment of parental distress, parent-child dysfunctional interaction, and difficult child. Reliability of the PSI was demonstrated to be in the 0.75–0.88 range with a high degree of internal consistency (Abidin, 2012), and found to be reliable and valid in a high-risk sample of families with toddlers (Barroso et al., 2016).

#### Secondary feasibility and acceptability outcomes

Feasibility was indexed by the proportion of families completing both intervention sessions, and study retention (Leon et al., 2011). Retention was assessed by examining the proportion of families assigned to the intervention condition who completed the 12-, 15-, and 24-month assessments.

Caregiver acceptability of the bedtime routine intervention was assessed at post-intervention based on percent implementing the bedtime routine components and using the Treatment Evaluation Inventory-Short Form (Kelley et al., 1989) adapted for this intervention (e.g., “I liked having a bedtime routine for my child,” “Overall, I have a positive reaction to the 3Cs for Bedtime ZZZs program.”). The seven items from this form were rated on a five-point Likert scale ranging from “strongly disagree” to “strongly agree.”

### Statistical analyses

Descriptive analyses (means and frequencies) were used to describe sociodemographic and bedtime routine data at 12 months (baseline), primary and secondary intervention outcomes at 15- and 24-month well visits, and intervention feasibility and acceptability at 15- and 24-month visits. *T*-tests for continuous variables and chi-square tests for categorical variables were used to examine missingness by group (intervention vs. usual care), baseline (12-month) family sociodemographic characteristics, bedtime routine frequency, and bedtime routine consistency. Only group assignment was associated with missingness (χ^2^ = 6.0, *p* = 0.014), such that those assigned to usual care were more likely to have missing data at any time point (52.0%) compared to those in the intervention condition (28.0%).

For bedtime routine frequency, the only continuous outcome measured at 12-, 15-, and 24-month visits, we used a repeated measures mixed effects model with maximum likelihood estimation to examine differences by group (intervention vs. usual care) in bedtime routine frequency at each time point. Chi-square tests were used to compare differences by group in bedtime routine consistency (0 = 4 or fewer nights/week, 1 = 5 or more nights/week) and the inclusion of specific activities encouraged in the intervention condition (bath, brushing teeth, reading) at 15- and 24-month visits. Correlations were conducted to explore bedtime routine frequency across time-points and by group. Because primary and secondary sleep (BISQ-R), social-emotional, and caregiver stress outcomes were assessed at 15 and 24 months only, ANOVAs by group (intervention vs. usual care) for continuous outcomes and chi-square analyses by group for categorical outcomes were conducted to examine outcomes at each time point, with missingness handled via listwise deletion.

Exploratory analyses to examine variation in bedtime routine frequency and consistency by group and caregiver educational attainment (high school education or less vs. some college or more) were also conducted. A group by time interaction by caregiver educational attainment interaction was included in the mixed effects model for bedtime routine frequency. For all other outcomes at 15 and 24 months, correlations, chi-square tests and two-way ANOVAs by group and caregiver educational attainment were examined. Analyses were conducted using STATA version 17 and IBM SPSS version 28.0 with a significance level *p* < 0.05 for main effects comparing intervention vs. usual care conditions, and *p* < 0.10 for analyses examining variation by educational attainment, given the exploratory nature of the analyses and the limited sample size for detecting interaction effects (Durand, 2013).

## Results

### Bedtime routines

Table 2 summarizes bedtime routine frequency (nights per week) and consistency by intervention vs. usual care groups, as well as by caregiver educational attainment within each group. The mixed effects model indicated there were no differences by group in bedtime routine frequency at baseline (estimate = 0.49, *z* = 1.46, *p* = 0.146). Group by time interaction effects were also non-significant at 15-month visits (estimate = 0.35, *z* = 0.75, *p* = 0.456) and 24-month visits (estimate = 0.34, *z* = 0.70, *p* = 0.486), indicating no group differences in change in bedtime routine frequency at these timepoints. Chi-square tests similarly showed no significant differences by group (at *p* < 0.05) in bedtime routine consistency at baseline (χ^2^ = 0.93 *p* = 0.335), 15-month visits (χ^2^ = 0.40, *p* = 0.530) or 24 months (χ^2^ = 2.92, *p* = 0.087).

**Table 2:** Bedtime routine frequency and consistency by group and caregiver educational attainment at each time point.

**Outcomes**	**Group**		**Group by caregiver educational attainment**			
	**Intervention**	**Usual care**	**Intervention**		**Usual care**	
			>**HS**	≤ **HS**	>**HS**	≤ **HS**
**Bedtime routine frequency, M (SD)**						
12 months	4.52 (2.43)	5.18 (2.17)	5.22 (2.18)	4.07 (2.51)	5.65 (1.69)	4.70 (2.51)
15 months	5.43 (1.85)	5.71 (1.54)	5.50 (2.07)	5.38 (1.74)	6.25 (1.12)	5.11 (1.74)
24 months	5.43 (1.43)	5.85 (1.68)	5.27 (1.30)	5.41 (1.52)	5.60 (1.80)	6.17 (1.53)
**Bedtime routine consistency (%)**						
12 months	52.2%	62.5%	61.1%	46.4%	70.0%	55.0%
15 months	70.0%	76.3%	68.8%	70.8%	90.0%	60.1%
24 months	66.7%	85.2%	80.0%	59.3%	93.3%	75.0%

Consistent bedtime routine = 5 nights per week or more; HS, high school; lower educational attainment = high school education or less.

The mixed effects model for bedtime routine frequency that explored potential moderating effects of caregiver educational attainment showed no significant interaction effects (at *p* < 0.10) at 15-month visits (estimate = 1.32, *z* = 1.41, *p* = 0.159) or at 24-month visits (estimate = −0.10, *z* = −0.10, *p* = 0.917). Exploratory chi-square analyses comparing intervention effects within each educational group also showed no significant differences (all *p*s > 0.10), indicating no moderating effects of educational attainment.

Interestingly, there were significant relationships between the number of nights per week a family engaged in a bedtime routine across the three time points (12-, 15-, and 24-month visits) for the intervention group, *r* = 0.36–0.59, *p* < 0.05, but not for the usual care group (see Supplementary Table 1). That is, the number of nights that a family engaged in a bedtime routine at 12 months was positively correlated with the number of nights at 15- and 24-month visits, but only for those in the intervention group. Furthermore, these relationships in the intervention group were stronger for those with lower educational attainment, *r* = 0.43–0.76, *p* < 0.05.

### Toddler sleep outcomes

As presented in Table 3, there were no differences between groups (intervention vs. usual care) for sleep outcomes at 15- and 24-month visits. Furthermore, there were no main effects for caregiver-perceived sleep problems or bedtime difficulties at either time point, *p* > 0.05. Perception of sleep problems ranged from 5.3 to 14.8%.

**Table 3:** Sleep, social-emotional, and caregiver outcomes at 15 and 24 months by group.

**Outcomes**	**Intervention M (SD)**	**Usual care M (SD)**	***F***	***p***	**ES**
**15 months**					
Bedtime (h)	21.21 (1.14)	20.95 (1.08)	1.06	0.307	0.01
SOL (min)	33.75 (28.91)	36.05 (35.81)	0.10	0.755	0.001
NW number	0.80 (0.87)	0.89 (0.98)	0.19	0.67	0.002
NW duration (min)	14.58 (13.67)	16.04 (25.37)	0.06	0.81	0.001
Longest sleep period (min)	565.74 (139.23)	601.85 (117.91)	1.50	0.225	0.020
Nighttime sleep (min)	609.22 (94.68)	620.82 (89.56)	0.31	0.580	0.004
Waketime	8.27 (1.59)	7.74 (1.14)	2.85	0.095	0.036
Total (24-h) sleep (h)	711.19 (98.70)	722.13 (94.26)	0.24	0.623	0.003
BITSEA externalizing	2.66 (2.60)	2.45 (2.86)	0.13	0.726	0.002
BITSEA internalizing	3.26 (2.27)	3.65 (3.00)	0.47	0.496	0.006
BITSEA dysregulation	3.41 (2.10)	3.48 (2.58)	0.02	0.903	0.000
BITSEA maladaptive	0.48 (0.72)	0.48 (0.72)	0.00	0.983	0.000
Caregiver stress	66.68 (18.79)	65.46 (20.62)	0.09	0.779	0.001
	**%**	**%**	**Chi-square**	***p***	
Bedtime difficulties	20.0%	15.8%	0.235	0.628	
Perceived sleep problem	12.5%	5.3%	1.25	0.264	
	**Intervention M (SD)**	**Usual care M (SD)**	***F***	***p***	**ES**
**24 months**					
Bedtime	21.26 (1.05)	21.02 (1.12)	0.83	0.365	0.012
SOL (min)	49.17 (34.41)	44.44 (38.14)	0.28	0.596	0.004
NW number	0.79 (1.00)	0.67 (1.07)	0.22	0.641	0.003
NW duration (min)	8.10 (5.36)	12.50 (17.12)	1.21	0.280	0.038
Longest sleep period (min)	590.32 (116.21)	600.13 (160.49)	0.09	0.769	0.001
Nighttime sleep (h)	596.13 (106.31)	622.48 (123.63)	0.89	0.349	0.013
Waketime	7.95 (1.69)	7.87 (1.33)	0.04	0.844	0.001
Total (24-h) sleep (h)	686.52 (98.57)	715.61 (148.14)	0.85	0.360	0.014
BITSEA externalizing	2.35 (2.20)	2.83 (2.96)	0.50	0.481	0.009
BITSEA internalizing	3.58 (2.05)	4.00 (3.22)	0.38	0.542	0.006
BITSEA dysregulation	3.44 (2.18)	3.75 (3.52)	0.17	0.679	0.003
BITSEA maladaptive	0.33 (0.63)	0.54 (1.14)	0.83	0.368	0.014
Caregiver stress	64.62 (18.00)	69.63 (28.99)	0.69	0.368	0.012
	**%**	**%**	**Chi-square**	***p***	
Bedtime difficulties	23.8%	25.9%	0.04	0.842	
Perceived sleep problem	9.5%	14.8%	0.45	0.503	

BITSEA, Brief Infant-Toddler Social and Emotional Assessment.

Exploratory analyses were conducted to assess for interactions (group by educational attainment) at 15- and 24-month visits for all sleep pattern variables (see Table 4). There were significant interactions for number and duration of night wakings, such that, compared to intervention children of caregivers with lower educational attainment, usual care children with caregivers of lower educational attainment were awake more often at night (1.33 vs. 0.50–0.82 times) and for longer duration (22.33 vs. 5.56–16.50 min), *p* < 0.10. That is, no differences were found between the intervention and control groups among those with higher educational attainment; however, toddlers whose mothers had lower educational attainment had better sleep consolidation in response to the intervention. At 24-month visits, there were no interactions for any toddler sleep patterns. Furthermore, there were no interactions by group and educational attainment for caregiver-perceived sleep problems or bedtime difficulties at 15- and 24-month visits, *p* > 0.10.

**Table 4:** Sleep, social-emotional, and caregiver outcomes at 15 and 24 months by group and caregiver educational attainment.

**Outcomes**	**Intervention**		**Usual care**		**Interaction**		
	>**HS educ (*****n*** = **16) M (SD)**	≤ **HS educ (*****n*** = **24) M (SD)**	>**HS educ (*****n*** = **20) M (SD)**	≤ **HS educ (*****n*** = **18) M (SD)**	***F***	***p***	**ES**
**15 months**							
Bedtime (h)	21:20 (1.05)	21.08 (1.22)	20:51 (0.96)	21:04 (1.22)	0.63	0.429	0.008
SOL (min)	38.75 (34.91)	30.42 (24.36)	33.50 (33.95)	38.89 (38.56)	0.84	0.362	0.011
NW number	0.82 (0.88)	0.79 (0.88)	0.50 (0.61)	1.33 (1.14)	4.59	0.036^*^	0.058
NW duration (min)	16.50 (17.17)	13.21 (11.03)	5.56 (3.91)	22.33 (30.58)	2.91	0.095^+^	0.062
Longest sleep period (min)	604.01 (116.89)	540.23 (149.20)	600.27 (142.85)	603.52 (88.47)	1.27	0.264	0.017
Nighttime sleep (min)	611.49 (76.57)	607.71 (106.64)	607.71 (106.64)	620.23 (68.28)	33.81	0.951	0.000
Waketime	8:25 (1.45)	7:43 (1.42)	8:10 (1.71)	7:46 (1.16)	0.227	0.635	0.003
Total (24-h) sleep (h)	712.93 (78.44)	709.99 (112.39)	717.41 (115.60)	727.11 (67.95)	0.78	0.781	0.001
BITSEA externalizing	1.78 (2.07)	3.27 (2.78)	1.80 (2.07)	3.10 (3.40)	0.027	0.871	0.000
BITSEA internalizing	2.56 (2.36)	3.71 (2.12)	2.50 (1.57)	4.80 (3.64)	1.09	0.301	0.013
BITSEA dysregulation	2.83 (1.62)	3.79 (2.32)	2.40 (2.19)	4.55 (2.54)	1.53	0.220	0.018
BITSEA maladaptive	0.44 (0.62)	0.50 (0.79)	0.35 (0.67)	0.60 (0.75)	0.38	0.540	0.005
Caregiver stress	65.13 (19.00)	67.57 (18.96)	62.35 (18.47)	68.74 (22.71)	0.20	0.658	0.002
	**%**	**%**	**%**	**%**	**Chi-square**	***p***	
Bedtime difficulties	25.0%	16.7%	5.0%	27.8%	0.24	0.628	
Perceived sleep problem	18.8%	8.3%	5.0%	5.6%	1.29	0.264	
	>**HS educ (*****n*** = **15) M (SD)**	≤ **HS educ (*****n*** = **27) M (SD)**	>**HS educ (*****n*** = **15) M (SD)**	≤ **HS educ (*****n*** = **12) M (SD)**	***F***	***p***	**ES**
**24 months**							
Bedtime	21:10 (0.65)	21:19 (1.23)	20:55 (1.14)	21:09 (1.14)	0.02	0.884	0.000
SOL (min)	50.67 (33.32)	48.33 (35.60)	40.00 (37.03)	50.00 (40.40)	0.46	0.502	0.007
NW number	0.47 (0.64)	0.96 (1.13)	0.33 (0.49)	1.08 (1.44)	0.25	0.616	0.004
NW duration (min)	8.33 (6.06)	800 (5.28)	6.00 (4.18)	17.14 (21.58)	1.93	0.175	0.062
Longest sleep period (min)	600.31 (101.41)	584.77 (125.18)	632.16 (103.62)	560.09 (209.93)	0.69	0.409	0.011
Nighttime sleep (h)	580.23 (125.47)	604.87 (95.51)	628.17 (108.34)	615.34 (145.21)	0.42	0.519	0.006
Waketime	7:22 (1.45)	8:16 (1.76)	7:35 (1.29)	8:14 (1.36)	0.11	0.742	0.002
Total (24-h) sleep (h)	694.68 (114.67)	681.55 (89.78)	715.95 (102.26)	715.20 (194.74)	0.04	0.850	0.001
BITSEA externalizing	2.45 (2.88)	2.30 (1.87)	2.14 (2.66)	3.80 (3.22)	1.67	0.201	0.030
BITSEA internalizing	3.38 (2.50)	3.70 (1.79)	2.64 (1.78)	5.90 (3.87)	5.17	0.027^*^	0.085
BITSEA dysregulation	3.15 (2.23)	3.61 (2.19)	1.71 (1.20)	6.60 (3.75)	12.14	<0.001^***^	0.178
BITSEA maladaptive	0.15 (0.55)	0.43 (0.66)	0.07 (0.27)	1.20 (1.55)	3.84	0.055^+^	0.064
Caregiver stress	64.27 (18.21)	64.86 (18.28)	60.43 (19.16)	82.50 (36.08)	3.29	0.075^+^	0.055
	**%**	**%**	**%**	**%**	**Chi-square**	***p***	
Bedtime difficulties	26.7%	22.2%	20.0%	33.3%	0.04	0.842	
Perceived sleep problem	0.0%	14.8%	0.0%	33.3%	0.45	0.503	

BITSEA, Brief Infant-Toddler Social and Emotional Assessment; Educ, education; HS, high school; lower educational attainment = high school education or less.

^+^*p* < 0.10, ^*^p < 0.05, ^***^p < 0.001.

### Toddler social-emotional outcomes

**Figure 3:**
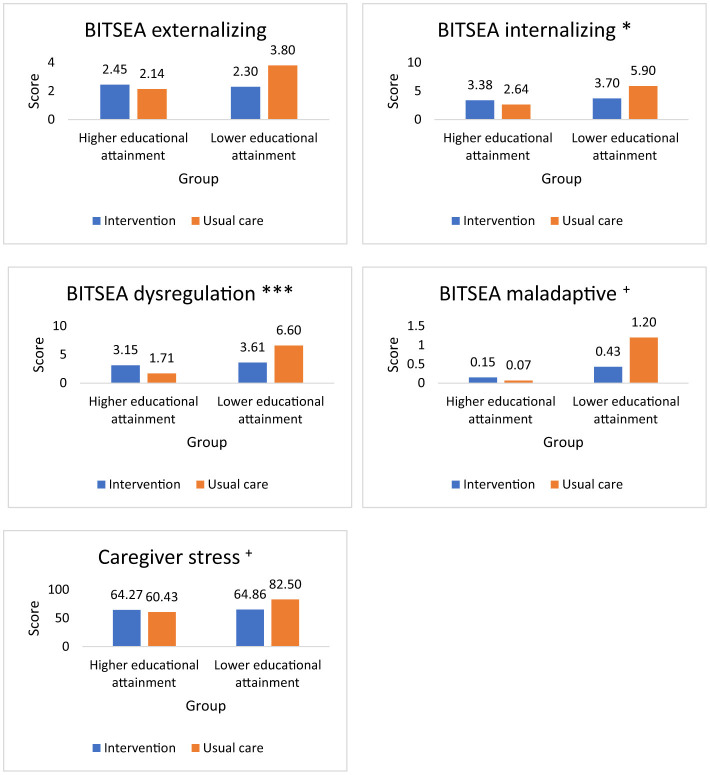
Brief Infant-Toddler Social and Emotional Assessment (BITSEA) and caregiver stress outcomes by intervention condition and caregiver educational attainment. ^+^*p* < 0.10, *p < 0.05, ***p < 0.001 for interaction of group x caregiver educational attainment.

As presented in Table 3, no significant group differences (intervention vs. usual care) were found across all social-emotional outcomes at 15- or 24-month visits, *p* > 0.05. In addition, no significant interaction effects were found (see Table 4). However, at 24-month visits, significant group by educational attainment interactions were observed for all subscales of the BITSEA except for externalizing, *p* < 0.10 (see Figure 3). For all these 24-month variables, minimal differences were found for high educational attainment families between the intervention and control groups. However, toddlers of caregivers with lower educational attainment had significantly better social-emotional outcomes in response to the intervention, on par with those obtained for toddlers of caregivers with higher educational attainment.

### Caregiver stress

Similar results were found for caregiver stress (see Tables 3, 4). There were no main effects at 15- and 24-month visits, nor an interaction at 15-month visits. However, differences were noted at 24 months, with an interaction between group (intervention vs. usual care) and educational attainment, in that caregivers with lower educational attainment in the control group reported significantly higher parental stress (*M* = 82.50), with no other differences noted (see Figure 3), *F*_(1, 57)_ = 3.29, *p* = 0.075. Similar lower levels of stress were noted for the high education families irrespective of group assignment (*M* = 64.27 and 60.43), as well as for caregivers with lower educational attainment in the intervention group (*M* = 64.86). Thus, there was an intervention effect on parental stress for toddlers of caregivers with lower educational attainment.

### Intervention feasibility and acceptability

**Figure 4:**
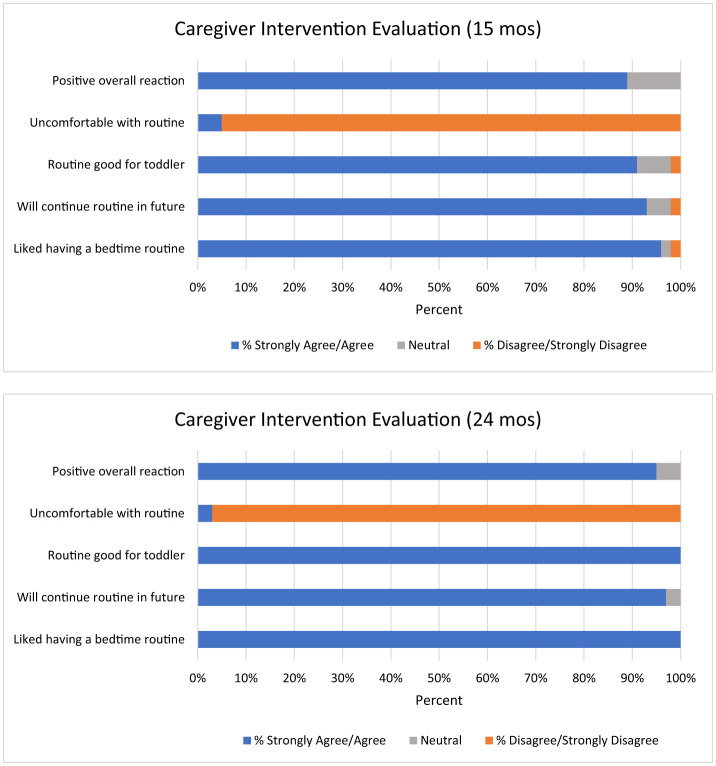
Intervention acceptability at 15 and 24 months.

Of the 46 caregiver-child dyads randomized to the intervention group, 85% engaged in both intervention sessions (at 12- and 15-month visits) and 83% completed both full assessments (15- and 24-month visits, see Figure 3). Ratings of intervention acceptability were strong (Figure 4). At 15-month visits, almost all caregivers “agreed” or “strongly agreed” that they felt positive about the intervention program and that they liked the strategies (95.5%), would use them in the future (93.2%), and that it was both good for their child (90.9%) and helped their child sleep (88.6%). Only one caregiver reported feeling uncomfortable with the routine (4.5%). Similar results were reported at 24-month visits, with 94.6% of the caregivers positive about the program, and all (100%) caregivers liking the routine, believing the routine was good for their child and helped their child sleep. One caregiver reported that the routine made them uncomfortable (2.7%). No differences were found in acceptability across levels of caregiver educational attainment, *p* > 0.05.

In the intervention condition, all families were encouraged to include a bath, brushing teeth, and reading as part of their child's bedtime routine. At 15-month visits, there were significant differences in inclusion of these activities based on group assignment for reading (75.0% intervention vs. 52.6% usual care; *p* = 0.040), with no differences in inclusion of brushing teeth (67.5 vs. 47.4%; *p* = 0.072) or a bath (90.0 vs. 89.5%, *p* = 0.939). No differences were found for bedtime routine activities at 24-month visits, *p* > 0.05. Across groups, 76.4% included a bath, 50.0% brushed teeth, and 47.2% read books at 24-month visits.

## Discussion

Overall, this pilot RCT found that a bedtime routine intervention was beneficial for families with lower educational attainment, as well as feasible and acceptable across all caregivers in the intervention condition. Although we did not find support for the overall impact of a bedtime routine intervention across all families randomized to this condition, the provision of bedtime routine support and materials was beneficial for families with lower levels of educational attainment. In these child-caregiver dyads, the bedtime routine intervention was found to positively impact sleep consolidation, social-emotional outcomes, and caregiver stress, primarily at 24 months of age.

Caregivers were overwhelmingly positive about the bedtime routine intervention. Almost all reported liking the routine and the overall intervention, believing it benefited their toddler, and that they planned to continue implementing the routine in the future. Furthermore, caregivers with lower educational attainment also found the routine to be acceptable. In partial support of hypotheses, the bedtime routine intervention resulted in increased sleep consolidation, better social-emotional functioning, and decreased caregiver stress, although only for caregivers with lower educational attainment, which is consistent with previous studies (Lam et al., 2023; Mindell et al., 2009). Of note, the bedtime routine intervention was associated with sleep consolidation, specifically decreased number and duration of night wakings, among toddlers of caregivers with lower educational attainment. Thus, a bedtime routine may have broader effects on sleep beyond just at bedtime, especially for families with lower educational attainment, consistent with the results seen in previous studies (Mindell et al., 2017).

The intervention was also associated with improved social-emotional functioning for toddlers of caregivers with lower educational attainment. At 24 months, social-emotional outcomes in toddlers of caregivers with lower educational attainment were on par with toddlers with a caregiver with higher educational attainment. These results are supported by past studies indicating a relationship between bedtime routines and better daytime behavior (Lam et al., 2023; Rijlaarsdam et al., 2016). This relationship may reflect the regularity and predictive nature of routines and positive caregiver-child interactions during the bedtime routine (Bobbitt and Gershoff, 2016; Mindell and Williamson, 2018). These findings support the contention that a bedtime routine can be not only associated with improved sleep, but also overall wellbeing in families with young children (Mindell and Williamson, 2018).

There are several possible reasons for the lack of an overall effect of the bedtime routine intervention across all families, which is contrary to our previous studies (Mindell et al., 2018, 2009). First, previous studies have primarily only included infants and toddlers with a caregiver-identified sleep problem, whereas we included all families presenting for a well-child visit in our study. Second, the majority of families across both groups reported instituting a bedtime routine at least five or more nights per week at baseline (57%), which increased to 74% by 24 months. By contrast, in prior bedtime routine research, most families do not report having a consistent bedtime routine at baseline (Mindell et al., 2018, 2009). This high overall rate may be specific to this primary care setting, although it is unknown what percentage of infant and toddler well-child visits regularly include a discussion of bedtime routines. Although the overall rate of implementation of a bedtime routine was high across all participants, caregivers with lower educational attainment reported engaging in a bedtime routine fewer nights per week at 12 months and were less likely to engage in a consistent bedtime routine (5 or more nights per week) at 24 months, across conditions.

The most salient finding from this work is that the intervention was beneficial for toddlers of caregivers with lower educational attainment, specifically a high school education or less. These results align with prior research showing lower rates of bedtime routine implementation in families with lower educational attainment (Hale et al., 2009; Williamson and Mindell, 2020), and advance the field by demonstrating an initial intervention benefits for those with lower educational attainment. This finding is particularly important in the context of well-documented pediatric sleep health disparities (Jackson et al., 2020; Lupini and Williamson, 2023). It may be that sleep education and resources provide additional support for both implementing a bedtime routine and developing one that incorporates literacy benefits, such as the inclusion of reading. One study found that a key barrier to establishing and maintaining optimal bedtime routines in families with young children was lack of knowledge and sources of information, whereas a facilitator included access to resources (Kitsaras et al., 2021). Thus, caregiver educational attainment may be a moderating socioeconomic factor when considering for whom a bedtime routine intervention may be most efficacious.

Finally, the bedtime routine intervention focused on three evidence-based bedtime routine components: connect, comfort, and calm. Although these aspects were not specifically assessed, the positive outcomes of improved child social-emotional outcomes and reduced caregiver stress among those with lower educational attainment may directly reflect these components. Alternatively, bedtime routines may result in reduced bedtime resistance and improved sleep consolidation, and in turn, result in these additional family benefits. Future studies should directly evaluate the importance of the individual components of this multi-component intervention, as well as the mechanisms underlying these changes. Furthermore, while there were no differences in the likelihood of having a bedtime routine across groups, approximately 25% more families in the intervention group included reading, although these differences were only observed at 15 months of age but not at 24 months. Interestingly, all families were less likely to engage in reading at 24 months compared to 15 months, even though these activities are likely even more important for early school readiness (Sells and Mendelsohn, 2021; Szumlas et al., 2021). Thus, future studies should consider ways to achieve sustained reading throughout toddlerhood and into the preschool years.

Primary care providers should consider encouraging all caregivers of young children to institute a consistent bedtime routine, especially caregivers of lower educational attainment. The number of nights a family engaged in a routine at 12 months was positively correlated with the number of nights at 15 and 24 months, but only for those in the intervention group. These results suggest that directly discussing a bedtime routine with families encourages implementation more nights per week. In addition, direct discussion, and in our case providing material support in the provision of additional books, bath time products, toothbrushes, and resource materials, resulted greater likelihood of engaging in reading at bedtime. Primary care providers within larger medical settings may be able to provide such material resources for many families. The bedtime routine charts can be simple paper handouts, some of which are freely accessible through public online resources (Mindell et al., 2021). Associated pediatric dental practices can often supply toothbrushes and information on the importance of starting dental hygiene at a young age. Programs such as *Reach Out and Read*, or annual book drives, can provide age-appropriate bedtime stories. Interestingly, one study found that the more books provided, the better children are prepared for kindergarten (Szumlas et al., 2021). Thus, more education and resource provision are better.

### Limitations

There are a number of limitations of the current study. First, this was a pilot RCT focused on initial intervention outcomes and conducted in the real-world setting of a primary care practice, with few exclusion criteria. Many families across intervention and usual care groups were already implementing a bedtime routine and continued to do so at both follow-up time points. Thus, we may not have had the power to detect statistically significant differences in outcomes or exploratory moderation analyses. However, we were able to generate preliminary information regarding clinically meaningful outcomes and demonstrate strong intervention feasibility and acceptability, all of which will inform future large-scale research that can fully evaluate the effectiveness of a bedtime routine (Leon et al., 2011). In addition, even with many families already implementing a bedtime routine, changes were found in the inclusion of reading for all intervention families and the intervention was efficacious for toddlers of caregivers with lower educational attainment across multiple outcomes. Second, as this pilot RCT was conducted in a real-world and thus highly generalizable setting, there were limitations that may have impacted the outcomes, such as the inability to randomize by family rather than by day of the week.

We also relied on caregivers' report regarding bedtime routines, sleep, and their child's social-emotional development, which may be subject to bias and shared method variance. Future research may benefit from adopting a multi-informant approach and more objective measures (e.g., behavioral observations) to assess both interactions and activities at bedtime, as well as outcomes. Additionally, the sleep, socio-emotional and caregiver stress measures were only administered at the 15- and 24-month visits, which limited our ability to examine changes from baseline and detect changes in outcomes across multiple timepoints. A larger and fully-powered future trial should include baseline measures to better understand intervention effects over time, as well as a longer follow-up period. Furthermore, caregivers were not compensated for their participation. The lack of compensation may have contributed to the relatively high attrition rate, especially for the usual care group, who were not provided all of the materials distributed in this study. Finally, to reduce participant burden, this study only included a narrow range of child and family outcomes and relied on brief measures. Future work should include measures of caregiver-child interactions at bedtime and broader child development and family functioning (e.g., school readiness, caregiver-child relationships), as well as caregiver stress and mental health functioning.

It is also important to note that this study was conducted during the COVID-19 pandemic. There were limitations imposed by the pandemic in terms of recruitment (e.g., ability to recruit across all days of the week, randomization by family) based on the restrictions in place within the primary care practice. The pandemic may have also impacted family retention across the year-long study. However, it is impressive that the intervention was found to be highly acceptable and feasible for all families, and possible within an urban primary care practice, even during these stressful times. The finding that a bedtime routine intervention can be implemented under difficult circumstances is encouraging and suggests that bedtime routines should be considered for families in all situations including times of stress (e.g., natural disasters, encampments), although future research in this regard is needed.

### Conclusion

This pilot study is among the first to examine the outcomes of a bedtime routine intervention in toddlers presenting to a real-world setting and to explore variation in outcomes according to socioeconomic factors. Conducting intervention research with the populations and in the settings identified for future scaling is crucial even in the earliest phases of research to ensure feasibility and avoid perpetuating healthcare disparities (Baumann and Cabassa, 2020; Weisz, 2014). Caregiver educational attainment was found to moderate intervention effects, in that a multi-component bedtime routine intervention for toddlers was found to improve sleep consolidation, social-emotional development, and caregiver stress only for toddlers of caregivers with lower educational attainment. The intervention resulted in increased inclusion of reading, a developmentally important activity, in the bedtime routine at 15 months, for all families. These findings highlight the promise of a consistent bedtime routine as an intervention to promote toddler development and family functioning, beyond established sleep benefits. Furthermore, a bedtime routine intervention is feasible and acceptable for families being seen in an urban primary care setting. Given that sleep health disparities begin in early childhood (Jackson et al., 2020; Lupini and Williamson, 2023), and the importance of bedtime routines for positive child outcomes (Mindell and Williamson, 2018), clinicians should consider discussing how to incorporate a simple, adaptive bedtime routine during well-child visits with all families.

## Data Availability

The datasets presented in this article are not readily available because deidentified individual participant data will be made available upon approval of outside requests by the study team and with appropriate human subjects permissions. Requests to access the datasets should be directed to jmindell@sju.edu.
