# Supplementary material for: A pilot bedtime routine intervention for toddlers in primary care: variation by caregiver educational attainment
Source: Front Sleep. 2026 Jan 6;4:1722530. doi: 10.3389/frsle.2025.1722530 (PMC12815716; doi:10.3389/frsle.2025.1722530)
Supplement: Supplementary file 1 [file Table_1.docx]

**Supplemental Table 1**

*Correlations showing engagement in a bedtime routine across time by intervention group and caregiver educational attainment*

| Group and educational attainment | Nights per week at 12 mos with nights per week at 15 mos | Nights per week at 15 mos with nights per week at 24 mos | Nights per week at 12 mos with nights per week at 24 mos |
| --- | --- | --- | --- |
|  |  |  |  |
| Intervention group (all) | .51 *** | .59 *** | .36 * |
| Lower educational attainment | .59 ** | .51* | .29 |
| Higher educational attainment | .22 | .43 | .27 |
|  |  |  |  |
| Usual care group (all) | .28 | .23 | .34 |
| Lower educational attainment | -.03 | .61 | .56 |
| Higher educational attainment | .15 | -.11 | .33 |

* *p* <.05, ** *p* <.01, *** *p* < .001
